# A Fast and Validated HPLC Method for Simultaneous Determination of Dopamine, Dobutamine, Phentolamine, Furosemide, and Aminophylline in Infusion Samples and Injection Formulations

**DOI:** 10.1155/2021/8821126

**Published:** 2021-02-27

**Authors:** Fuchao Chen, Baoxia Fang, Sicen Wang

**Affiliations:** ^1^School of Pharmacy, Xi'an Jiaotong University, Xi'an 710061, Shanxi, China; ^2^Sinopharm Dongfeng General Hospital, Hubei University of Medicine, Shiyan, Hubei 442008, China

## Abstract

A simple, fast, and validated HPLC method was developed for the simultaneous quantization of five cardiovascular agents: dopamine (DPM), dobutamine (DBM), phentolamine (PTM), furosemide (FSM), and aminophylline (APL) either in infusion samples or in an injection dosage form. The proposed method was achieved with a 150 mm × 4.6 mm, 5.0 *μ*m C_18_ column, by using a simple linear gradient. Mobile phase A was buffer (50 mM KH_2_PO_4_) and mobile Phase B was acetonitrile at a flow rate of 1.0 mL/min. The column temperature was kept at 30°C, and the injection volume was 20 *μ*L. All analytes were separated simultaneously at a retention time (tr) of 3.93, 5.84, 7.06, 8.76, and 9.67 min for DPM, DBM, PTM, FSM, and APL, respectively, with a total run time of less than 15.0 min. The proposed method was validated according to ICH guidelines with respect to accuracy, precision, linearity, limit of detection, limit of quantitation, and robustness. Linearity was obtained over a concentration range of 12.0–240.0, 12.0–240.0, 20.0–200.0, 6.0–240.0, and 10.0–200.0 *μ*g/mL DPM, DBM, PTM, FSM, and APL, respectively. Interday and intraday accuracy and precision data were recorded in the acceptable limits. The new method has successfully been applied for quantification of all five drugs in their injection dosage form, infusion samples, and for evaluation of the stability of investigated drugs in mixtures for endovenous use. The results of the stability study showed that mixtures of DPM, DBM, PTM, FSM, and APL in 5% glucose or 0.9% sodium chloride injection were stable for 48 hours when stored in polypropylene syringes at 25°C.

## 1. Introduction

**Figure 1:**
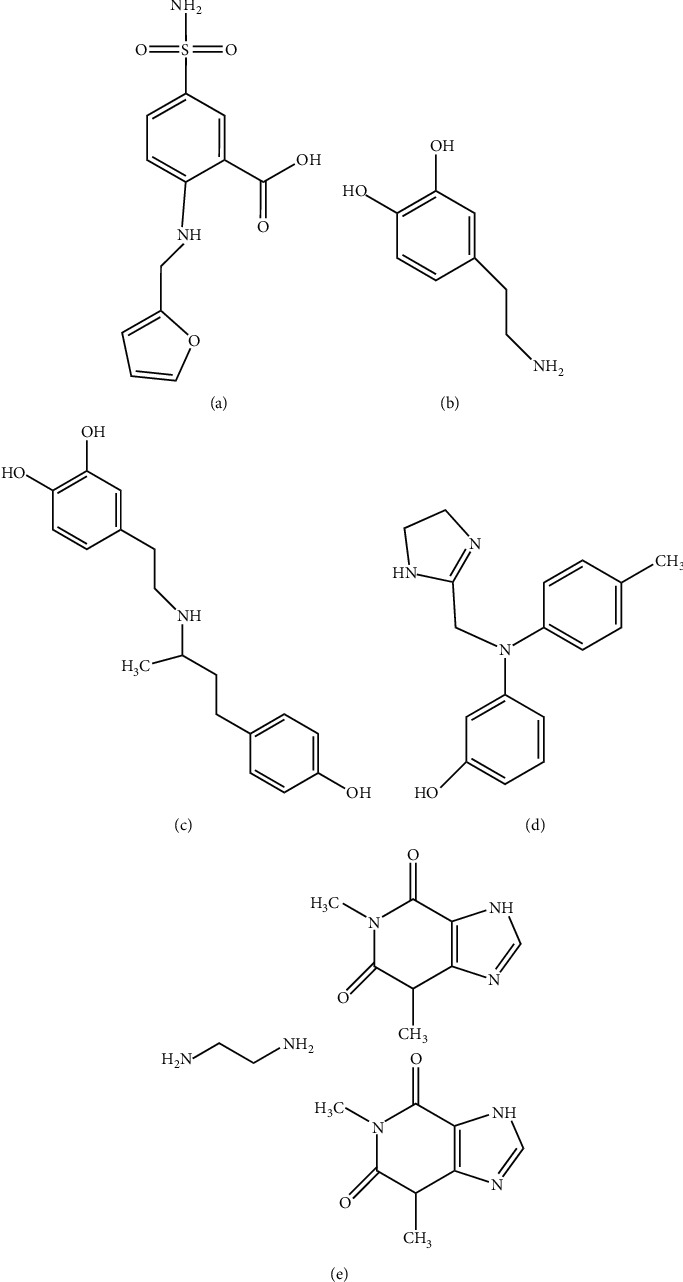
Chemical structure of FSM (a), DPM (b), DBM (c), PTM (d), and APL (e).

Congestive heart failure (HF) is an important health care problem across the world [1]. The prevalence of HF is 1-2% in developed countries and is expected to rise even further in the next decades [2, 3]. Diuretic therapy is an essential part of the management of the majority of patients with HF [4]. Furosemide (FSM, Figure 1(a)), 4-chloro-2-((furan-2-ylmethyl) amino)-5- sulfamoylbenzoic acid, a loop diuretic drug, is frequently administered to increase urinary output [5–10]. However, diuretic resistance is common in patients with acute HF and is associated with an increased risk of morbidity and mortality. The current strategies to overcome diuretic resistance include restriction of sodium intake, coadministration regimens, continuous diuretic infusions, and mechanical ultrafiltration [11, 12].

Dopamine (DPM, Figure 1(b)), 2-(3, 4-dihydroxyphenyl) ethylamine, is an endogenous substance with dose-dependent effects. Dobutamine (DBM, Figure 1(c)), 4-(2-(3-(4-hydroxy- phenyl)-1-methyl-propylamino)-ethyl)-benzene-1,2-diol, a beta-1 agonist catecholamine has cardiac stimulant action without evoking vasoconstriction or tachycardia. Phentolamine (PTM, Figure 1(d)), 3-((4, 5-dihydro-1H-imidazol-2-ylmethyl) (4-methylphenyl) amino) phenol, is a long-acting, adrenergic, nonselective alpha-receptor blocking agent. Aminophylline (APL, Figure 1(e)), bis(1, 3-dimethyl-2,3,6,7-tetrahydro-1H-purine-2,6-dione), ethane-1, 2-diamine, is the ethylenediamine salt of theophylline. It relaxes certain smooth muscles in the bronchi, produces diuresis, and causes an increase in gastric secretion. Previous guidelines and studies for HF management have suggested that the addition of DPM, DBM, PTM, and APL to diuretic therapy enhances decongestion and preserves renal function during diuretic therapy [13–18]. In addition, coadministration of diuretic therapy has become an accepted clinical practice for HF patients in our institution. However, there are no commercially available such drug mixtures, and they must be prepared in a centralized preparation service department for clinical use. Therefore, specific information on drug stability in various solutions, individually or associated, is necessary to ensure patient's safety. Analytical methods for their quality control are interesting not only as part of hospital-based quality control of the five cardiovascular agents in their injection dosage form but also for drug stability study and intravenous admixture preparation error monitoring.

By reviewing the literature, various analytical methods have been developed for individual quantification of DPM, DBM, PTM, FSM, or APL, or in combination with other drugs by HPLC [19–29]. Our study endeavors to develop and validate a reversed-phase HPLC method for simultaneous quantification of DPM, DBM, PTM, FSM, and APL in infusion samples and in the injection dosage form. To the extent of our knowledge, no analytical method based on reversed-phase HPLC has been reported so far for the simultaneous estimation of the mixture containing DPM, DBM, PTM, FSM, and APL. Therefore, the current study was aimed to develop a rapid, simple, and reproducible reversed-phase HPLC method for simultaneous quantification of the above five cardiovascular drugs in infusion samples and in injection dosage form and to determine the stability of the five cardiovascular drugs, diluted with 0.9% sodium chloride or 5% glucose injection and packaged in polypropylene syringes for 48 hours (2 days) at 25°C.

## 2. Materials and Methods

### 2.1. Chemicals and Reagents

The reference standards of DPM, DBM, PTM, FSM, and APL were procured from the National Institute for Control of Pharmaceutical and Biological Products (Beijing, PR China) and stored at 4°C. Potassium dihydrogen phosphate KH_2_PO_4_ (AR grade) was supplied by Shenzhen Yihao Technology Development Co., Ltd. (Guangdong, China). Water (HPLC -grade) was prepared in our laboratory using a flow water purification system (Millipore Corp., USA). Acetonitrile (HPLC-grade) was purchased from Fisher Scientific International (St Louis, MO, USA). The marketed formulations of drug used in this study were DPM hydrochloride injection 20 mg/2 mL (Harvest Pharmaceutical Co., Ltd., Shanghai, PR China), DBM hydrochloride injection 20 mg/2 mL (Shanghai No. 1 Biochemical and Pharmaceutical Co. Ltd., Shanghai, PR China), PTM mesylate injection 20 mg/2 mL (Beijing Novartis Pharmaceutical Co. Ltd., Beijing, PR China), FSM injection 20 mg/2 mL (Harvest Pharmaceutical Co., Ltd., Shanghai, PR China), and APL injection 20 mg/2 mL (Henan Lingrui Pharmaceutical Co., Ltd., Xinyang, PR China). The commercial solution of 0.9% sodium chloride injection was purchased from Kelun Pharmaceutical Co., Ltd. (Sichuang, China).

### 2.2. Instrumentation

The HPLC system used for the optimized method was a UltiMate3000 LC (Thermo Fisher) consisting of a column oven, a quaternary pump, an automatic injector, and a diode array detector. The system was controlled by Chromeleon 7.2 software (all from Thermo Fisher).

### 2.3. Chromatographic Conditions

A SinoChrom ODS-BP column (4.6  mm × 150  mm i.d. and 5.0 *μ*m particle size) was used as the stationary phase. Mobile phase A was 50 mM KH_2_PO_4_ buffer, and mobile phase B was acetonitrile with simple gradient program (0–5 min: MP-A: 95–65; 5–10 min: MP-A: 65–65; 10–11 min: MP-A: 65–95; 11–15 min: MP-A: 95–65) was delivered at a flow rate of 1.0 mL/min. The mobile phase was prepared daily and filtered with a 0.45 *μ*m membrane filter (Millipore Corp., USA). The column temperature was set at 30°C. And, samples were analyzed at a wavelength of 280 nm and injected at 20 *μ*L injection volume.

### 2.4. Preparation of Stock, Working, and Standard Curve Solutions

The standard stock solutions (0.6 mg/mL) of DPM, DBM, and FSM and (1.0 mg/mL) of PTM and APL were prepared by dissolving an accurately weighed 6 or 10 mg of each reference standards separately in 10 mL of deionised water in a 10 mL volumetric flask. The solutions were kept at 20°C until use.

For the linearity studies, a 6-point calibration curve was prepared by diluting the working standard solution with deionised water and the range of this calibration curve (from 12.0 to 240.0 *μ*g/mL for DPM, 12.0 to 240.0 *μ*g/mL for DBM, 20.0 to 200.0 *μ*g/mL for PTM, 6.0 to 240.0 *μ*g/mL for FSM, and 10.0 to 200.0 *μ*g/mL for APL).

### 2.5. Validation of Method

Method validation of quantitative analysis was performed on parameters such as linearity, precision, recovery, and specificity of the five drugs. The linearity of response was performed using plotting peak areas against the concentration of the injected standard. Method precision was evaluated based on intraday and interday variability. Intraday variability was conducted by injecting the triplicate determination of quality control samples (12. 0, 60.0, 120.0, and 240 *μ*g/mL for DPM and DBM; 6.0, 60.0, 120.0, and 240 *μ*g/mL for FSM, 20.0, 50.0, 100.0; and 200 *μ*g/mL for PTM, 10.0, 50.0, 100.0, and 200 *μ*g/mL for APL) six consecutive times in the same day. Interday variability was realized by using the same quality control samples for six successive days. The relative standard deviation (RSD) values were calculated for the integration area and considered to be the measure of precision. The limit of detection (LOD) for each marker compound was determined at signal-to-noise ratios (S/N) of 3. For limit of quantification (LOQ), the ratio considered was 10 : 1 with RSD % value less than 10% [30, 31]. Accuracy of the method was determined by addition of known amounts of DPM, DBM, PTM, FSM, and APL standard drugs (*n* = 3, at each level of 50, 100, and 150% levels) into infusion samples in triplicate. In this work, the mean recovery of the DPM, DBM, PTM, FSM, and APL concentration was 100 ± 2% for acceptance. The forced degradation studies were performed to prove the specificity of the HPLC method. The DPM, DBM, PTM, FSM, or APL samples were prepared with distilled water and later diluted with distilled water, aqueous hydrochloric acid, aqueous sodium hydroxide, or aqueous hydrogen peroxide solution for 5 h at 60°C. The chromatogram obtained for the degraded preparation was compared with a chromatogram obtained from the standard curve to determine whether or not any degrading peaks were produced and any changes in concentration, retention time, and peak shape of DPM, DBM, PTM, FSM, and APL. Robustness of the developed method was evaluated with respect to small deliberate alterations in the flow rate (1.0 ± 0.1 mL·min^−1^), the column temperature (30 ± 1°C), and the buffer concentration (50 ± 2 mM).

### 2.6. Application for Pharmaceutical Dosage Forms

This validated assay was used to quantify the amount of DPM, DBM, PTM, FSM, and APL in their commercially available injection dosage form. For test sample solution, an appropriate volume of the aliquot was transferred into 100 mL volumetric flask and diluted to the mark with diluent to obtain a test solution of DPM (0.12 mg/mL), DBM (0.12 mg/mL), PTM (0.10 mg/mL), FSM (0.12 mg/mL), and APL (0.05 mg/mL), respectively. The solution was filtered through a 0.45 *μ*m membrane filter.

### 2.7. Stability of Investigated Drugs in Mixtures

This study was carried out to mimic as closely as possible a projected routine use of the five cardiovascular agents. Commercially available DPM, DBM, PTM, FSM, or APL were mixed in 50 mL polypropylene syringes of 5% glucose or 0.9% sodium chloride injection at concentrations of 3.0 mg/mL, 3.0 mg/mL, 1.0 mg/ml, 4.0 mg/ml, and 10.0 mg/mL, respectively. All admixtures were investigated at room temperature (25°C) over 48 hours with light protection or light exposure. The stability studies were evaluated in triplicate for each type of infusion media and storage condition. The physical parameters such as color change, presence of turbidity, and precipitate were evaluated qualitatively whenever samples were withdrawn. At each time point, the pH values for each admixture were determined with a precision pH meter (model PHSJ-4F, INESA Scientific Instrument Co., Ltd., Shanghai, China). The concentrations of DPM, DBM, PTM, FSM, or APL were determined by the above described HPLC-DAD method. All samples from each syringe were analyzed in triplicate (total *n* = 3). The initial concentration of the five cardiovascular agents was defined as 100%, and subsequent sample concentrations for the drugs in the mixtures were reported as the percentage of the initial concentration. Drug stability was defined as the remaining 90% of the initial value of each drug.

## 3. Results and Discussion

### 3.1. Method Development and Optimization

**Figure 2:**
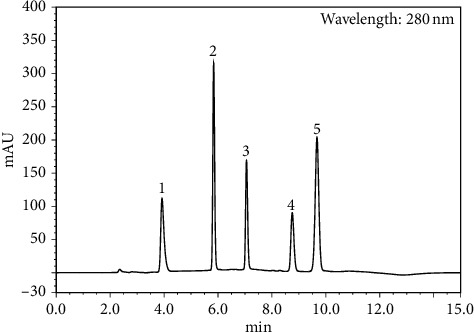
HPLC chromatograms of FSM (1), DPM (2), DBM (3), PTM (4), and APL (5).

**Figure 3:**
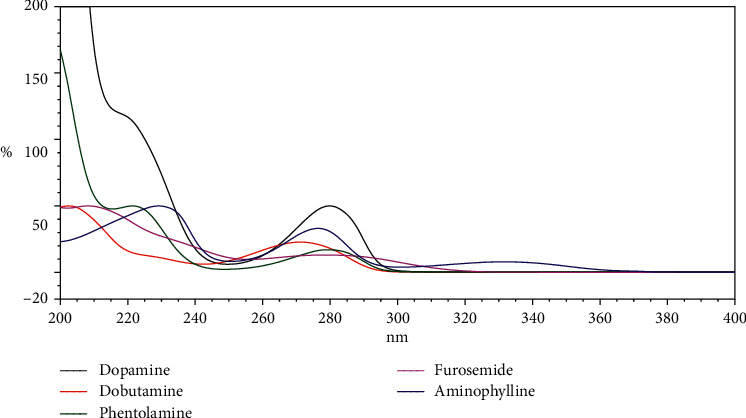
Overlaid UV absorption spectrum of FSM, DPM, DBM, PTM, and APL.

The widespread use of a diuretic drug of FSM and diuretic adjutants such as DPM, DBM, PTM, and APL for the management of congestive heart failure has stimulated our interest to develop a fast and simple analytical method for the simultaneous determination of the five cardiovascular agents in infusion samples and in injection formulations. With regard to the physical and chemical properties of the five cardiovascular agents and the information obtained from the literature, a series of trials of the current method was performed, such as different compositions of mobile phase, detection wavelength, sample preparation procedure, and column lengths, with different pH values and buffering agents. A high-quality separation and symmetric peak shape for all the tested analytes was achieved with mobile phase 50 mM KH_2_PO_4_ and acetonitrile with a simple linear gradient at 1 mL/min flow rate. Under the described conditions, the retention time for DPM, DBM, PTM, FSM, and APL was observed at 3.93, 5.84, 7.06, 8.76, and 9.67 min, respectively, as shown in Figure 2. DPM exhibits a *λ*_max_ at 280 nm, DBM *λ*_max_ at 271 nm, PTM *λ*_max_ at 279 nm, FSM *λ*_max_ at 279 nm, and APL *λ*_max_ at 276 nm (Figure 3). In order to increase the sensitivity for the analytes and applicability for routine work, a wavelength of 280 nm was selected for detection. The forced degradation peaks are well resolved from the corresponding drug and did not interfere with its determination.

### 3.2. Method Validation

Under the above described experimental conditions, the linearity of the proposed method was investigated by plotting the peak area DPM, DBM, PTM, FSM, and APL versus the concentration of each standard drug. The characteristic parameters for regression equations of the proposed HPLC method are given in Table 1. The values of LOD in the developed method were 0.48, 0.12, 0.20, 0.24, and 0.18 *μ*g/mL for DPM, DBM, PTM, FSM, and APL, respectively. The calculated LOQ values obtained were 1.80, 0.42, 0.70, 0.90, and 0.63 *μ*g/mL for DPM, DBM, PTM, FSM, and APL, respectively, with %RSD less than five (accepted criteria in less than 10%). The precision and accuracy data for both inter- and intraday analysis of DPM, DBM, PTM, FSM, and APL in the quality control samples at three levels is depicted in Table 2. The results of precision and accuracy were found to be within the acceptable limits and revealed that the new method is precise and accurate. Degradation was observed in different stress conditions, and the peaks for DPM, DBM, PTM, FSM, and APL were clearly separated from peaks for the degradation products.  Table 3 represents the assay and purity check for DPM, DBM, PTM, FSM, and APL under different stress conditions. The interference between each compound with degradation products can be accurately located to a certain extent by overall shape or 3D absorption spectra. The peak purity index for the active ingredients and degradants was found to be greater than 0.999, which proves that this method is specific in nature. The robustness results are displayed in Table 4. It was demonstrated that applied minor variations of the flow rate, column temperature, and buffer concentration did not affect the recovery of the five studied drugs.

### 3.3. Assay of Pharmaceutical Dosage Forms

The validated method was applied to the determination of DPM, DBM, PTM, FSM, and APL in the commercially available injection dosage form. The results are shown in Table 5 and are in good agreement with those obtained using the comparison methods described in the Chinese Pharmacopoeia (2015 edition) [32].

### 3.4. Stability of Investigated Drugs in Mixtures

The mean concentrations over time of DPM, DBM, PTM, FSM, and APL diluted with 5% glucose or 0.9% sodium chloride injection and packaged in polypropylene syringes are outlined in Tables 6 and 7, respectively. Defined as a decomposition of ≤10% compared to the initial concentration, DPM, DBM, PTM, FSM, and APL were stable in either 5% glucose or 0.9% sodium chloride injection and at each storage condition over the whole study period. After 48 hours, there were no visible signs of particulate matter in any of the polypropylene syringes. Each solution remained colorless over the course of the study. There were no major changes in pH for all of the clear solutions. The mean (±standard deviation) pH values were 3.71 ± 0.11, 3.55 ± 0.38, 4.70 ± 0.20, 9.11 ± 0.05, and 8.62 ± 0.06 for DPM, DBM, PTM, FSM, and APL stored in 5% glucose at 25°C, respectively. The mean (±standard deviation) pH values were 4.05 ± 0.19, 3.50 ± 0.29, 4.50 ± 0.23, 9.10 ± 0.05, and 8.88 ± 0.02 for DPM, DBM, PTM, FSM, and APL stored in 0.9% sodium chloride injection at 25°C, respectively.

## 4. Conclusion

The present investigation represents a simple and accurate HPLC method for the separation of DPM, DBM, PTM, FSM, and APL. The proposed method could be applied for the analysis of all five drugs in their injection dosage form, infusion samples, and for the evaluation of the stability of investigated drugs in mixtures for endovenous use. On the basis of the results of stability study, DPM, DBM, PTM, FSM, and APL mixed in 5% glucose or 0.9% sodium chloride injection were physically and chemically stable at 25°C for 48 hours (2 days) when stored in polypropylene syringes.

## Figures and Tables

**Table 1:** Analytical parameters for DPM, DBM, PTM, FSM, and APL.

Analytical parameter	DPM	DBM	PTM	FSM	APL
*λ*_max_ wavelength (nm)	280	271	279	279	276
Retention time(min)	3.93	5.84	7.06	8.76	9.67
Theoretical plate (mean ± SD)	5102 ± 86	34975 ± 1595	39586 ± 1724	27469 ± 1061	23985 ± 1362
Linear range (mg/L)	12.0 – 240.0	12.0 – 240.0	20.0 – 200.0	6.0 – 240.0	10.0 – 200.0
Linear equation	*Y* = 0.9892*X* − 2.0961	*Y* = 0.9672*X* + 0.9111	*Y* = 1.6493*X* + 1.4782	*Y* = 4.5218*X* + 32.275	*Y* = 2.9828X − 1.3333
Coefficient of correlation (r)	0.9999	0.9993	0.9995	0.9990	0.9998
Detection limit (mg/L)	0.48	0.12	0.20	0.24	0.18
Quantification limit (mg/L)	1.80	0.42	0.70	0.90	0.63

**Table 2:** Validation of HPLC method.

Compound	Measured concentrations (*μ*g/mL)	Accuracy (%)	Precision RSD (%)	
			Intraday	Interday
DPM hydrochloride	60.0	98.8	1.2	1.9
	120.0	102.1	0.7	1.4
	180.0	101.9	1.6	1.8

DBM hydrochloride	60.0	100.4	1.1	1.7
	120.0	100.2	0.3	0.8
	180.0	101.9	1.0	1.4

PTM mesylate	50.0	100.8	0.6	1.2
	100.0	101.3	0.2	1.1
	150.0	99.8	1.4	0.8

FSM	60.0	102.6	1.5	2.3
	120.0	99.4	0.9	0.5
	180.0	102.0	0.7	1.9

APL	50.0	98.5	1.2	1.6
	100.0	101.4	0.6	2.0
	150.0	99.9	0.7	2.5

**Table 3:** Summary of the forced degradation of DPM, DBM, PTM, FSM, and APL.

Compound	Stress condition	Degradation (%)	Peak purity index
DPM hydrochloride	Acidic	3.4	0.9992
	Alkaline	44.6	0.9999
	Oxidative	1.9	1.0000

DBM hydrochloride	Acidic	1.2	0.9998
	Alkaline	51.4	0.9999
	Oxidative	33.7	0.9999

PTM mesylate	Acidic	40.5	0.9998
	Alkaline	42.2	0.9995
	Oxidative	2.3	0.9999

FSM	Acidic	16.1	0.9998
	Alkaline	25.3	0.9999
	Oxidative	7.6	0.9999

APL	Acidic	1.1	1.0000
	Alkaline	4.5	0.9991
	Oxidative	10.6	0.9995

**Table 4:** The robustness of the proposed HPLC method.

Parameter	Variable	Recovery (%)				
		DPM	DBM	PTM	FSM	APL
Flow rate (mL·min^−1)^	0.9	97.8	98.8	98.4	99.9	98.9
	1.0	97.5	100.6	101.3	98.7	98.4
	1.1	100.3	98.1	98.5	100.1	99.7
	29	99.5	99.1	100.2	99.4	98.8
Column temperature (°C)	30	99.6	98.9	99.7	98.6	99
	31	98.8	99.2	99.7	100.5	99.4
	48	99.2	99.9	97.9	99.6	101.3
Buffer concentration (mM)	50	100.9	98.6	101.6	100.7	98.6
	52	98.7	98.3	98.3	98.9	100.5
Mean		99.1	99.1	99.5	99.6	99.4
%RSD		1.1	0.8	1.3	0.8	1.0

**Table 5:** The results obtained by the proposed methods and the reference methods.

Compound	The proposed methods		The Chinese Pharmacopoeia (2015)	
	Accuracy (%)	RSD (%)	Accuracy (%)	RSD (%)
DPM hydrochloride	100.7	1.1	100.1	0.8
DBM hydrochloride	97.8	1.2	98.7	0.5
PTM mesylate	98.2	0.8	99.4	0.6
FSM	101.6	0.4	99.8	1.1
APL	98.4	1.0	99.2	0.9

**Table 6:** Mean concentrations of DPM, DBM, PTM, FSM, and APL in 0.9% sodium chloride injection with light protection or light exposure at 25°C.

Storage conditions	Time (hours)	DPM	DBM	PTM	FSM	APL
Light protection	0	100.0 ± 1.3	100.0 ± 1.0	100.0 ± 0.6	100.0 ± 0.8	100.0 ± 1.4
	2	102.5 ± 1.9	100.3 ± 0.4	97.6 ± 2.2	100.1 ± 0.3	100.6 ± 0.2
	4	102.5 ± 0.8	99.8 ± 1.2	100.0 ± 0.2	98.7 ± 1.1	98.9 ± 1.1
	8	102.8 ± 0.4	101.7 ± 0.7	99.9 ± 0.4	98.8 ± 0.5	100.7 ± 0.9
	24	103.0 ± 0.5	100.9 ± 0.6	101.9 ± 1.1	99.7 ± 0.2	100.5 ± 0.5
	48	103.2 ± 1.4	99.1 ± 1.2	100.5 ± 0.8	97.8 ± 1.1	98.6 ± 0.4

Light exposure	0	100.0 ± 1.1	100.0 ± 0.4	100.0 ± 0.7	100.0 ± 0.2	100.0 ± 0.6
	2	102.0 ± 0.4	101.2 ± 0.9	100.9 ± 1.0	99.1 ± 0.4	100.7 ± 1.3
	4	102.2 ± 0.5	103.0 ± 2.3	99.8 ± 0.2	99.3 ± 0.7	101.0 ± 1.2
	8	101.5 ± 1.3	100.7 ± 0.8	100.6 ± 0.3	98.1 ± 1.0	97.7 ± 2.0
	24	101.8 ± 0.3	100.2 ± 0.5	97.6 ± 0.2	99.2 ± 0.8	98.9 ± 0.3
	48	102.3 ± 1.8	98.1 ± 1.4	96.2 ± 1.4	98.3 ± 1.3	98.4 ± 1.0

**Table 7:** Mean concentrations of DPM, DBM, PTM, FSM, and APL in 5% glucose injection with light protection or light exposure at 25°C.

Storage conditions	Time (hours)	DPM	DBM	PTM	FSM	APL
Light protection	0	100.0 ± 0.4	100.0 ± 0.2	100.0 ± 1.1	100.0 ± 0.6	100.0 ± 0.5
	2	100.8 ± 1.2	102.1 ± 1.1	99.5 ± 0.3	100.3 ± 0.2	98.4 ± 1.0
	4	101.6 ± 1.3	101.8 ± 0.6	100.2 ± 1.0	103.0 ± 1.8	99.3 ± 0.2
	8	102.0 ± 0.4	100.9 ± 0.6	101.1 ± 1.2	98.8 ± 2.1	98.7 ± 0.8
	24	101.7 ± 1.5	98.6 ± 1.7	98.8 ± 0.8	98.2 ± 0.8	99.1 ± 0.6
	48	101.6 ± 0.8	101.8 ± 1.3	98.7 ± 1.6	100.0 ± 0.3	98.4 ± 1.5

Light exposure	0	100.0 ± 0.5	100.0 ± 1.0	100.0 ± 0.5	100.0 ± 0.2	100.0 ± 0.7
	2	99.3 ± 1.6	101.6 ± 0.9	98.5 ± 1.9	98.0 ± 1.4	98.0 ± 1.1
	4	100.2 ± 0.9	101.9 ± 0.6	100.9 ± 0.2	98.8 ± 0.6	98.4 ± 0.8
	8	102.3 ± 1.1	99.8 ± 1.0	97.4 ± 2.1	99.4 ± 1.5	98.2 ± 0.4
	24	101.2 ± 0.7	101.8 ± 0.5	98.1 ± 1.7	98.9 ± 1.7	99.1 ± 0.2
	48	101.5 ± 1.1	103.3 ± 2.1	96.7 ± 1.0	97.5 ± 1.9	98.9 ± 1.3

## Data Availability

All data used to support the findings of this study will be made available from the corresponding author upon reasonable request.
